# Effects of γ-oryzanol on motor function in a spinal cord injury model

**DOI:** 10.1515/tnsci-2022-0310

**Published:** 2023-09-20

**Authors:** ZhiYi Fan, WanDa Zhan, Jun Cai

**Affiliations:** Clinical Medical College, Yangzhou University, 98 West Nantong Road, Yangzhou, Jiangsu, 225000, China

**Keywords:** spinal cord injury, γ-oryzanol, scar, intraperitoneal injection, motor function

## Abstract

**Objective:**

Spinal cord injury (SCI) is caused by disease or trauma and results in a partial or complete loss of motor or sensory function below the injury level. Most patients with SCI are young, and long-term disability imposes both psychological and financial burdens. Rice is the most abundant source of γ-oryzanol, which exhibits both antioxidant and anti-inflammatory properties. γ-Oryzanol has been shown to cross the blood–brain barrier in an intact form and have beneficial effects on brain function. To the best of our knowledge, this is the first study to report the effect of γ-oryzanol on motor function recovery in mice after SCI.

**Methods:**

Mice were randomly divided into three groups: the sham group, the injury group, and the γ-oryzanol-treated group that received an intraperitoneal γ-oryzanol (100 mg/kg) injection every 2 days for 42 days after SCI. The effect of γ-oryzanol was assessed through various approaches. Behavioral tests were performed using Basso mouse scale scores and gait analysis. Hematoxylin and eosin staining, Luxol fast blue staining, magnetic resonance imaging ,and immunofluorescence staining were used to observe the lesion area changes, demyelination, axonal regeneration, and scar tissue formation. The levels of inflammatory cytokines in the peripheral blood of mice were assessed by enzyme-linked immunosorbent assay.

**Results:**

Behavioral tests showed that γ-oryzanol treatment improved gait following SCI. Pathological examination revealed that demyelination at the site of injury improved with γ-oryzanol treatment and was accompanied by the retention of axons associated with motor function and reduced scarring. Additionally, γ-oryzanol treatment decreased the serum levels of pro-inflammatory factors.

**Conclusions:**

Studies have shown that γ-oryzanol promotes motor function recovery in mice after SCI. Therefore, γ-oryzanol might be the latent target for SCI therapy.

## 1 Introduction

Spinal cord injury (SCI) can cause irreversible sensory and motor dysfunction of the limbs of patients, which brings huge burden to the family and society [1]. In addition, current treatments for SCI have not achieved the expected effect [2,3].

Rice is the staple food worldwide [4,5]. Rice contains essential amino acids and dietary fiber and is rich in various bioactive phytochemicals, such as vitamin E (tocopherol and tocotrienol), γ-aminobutyric acid, anthocyanins, phenols, flavonoids, and γ-oryzanol [6,7]. Compared with other plant-based diets, rice is the most abundant source of γ-oryzanol. The name “γ-oryzanol” comes from rice because it was originally found in rice bran oil (“oryza-”) and has a hydroxyl group (“-nol”) [8].

γ-Oryzanol has been shown to be a safe product without major side effects [9,10]. Numerous studies have reported the beneficial health properties of γ-oryzanol, including anti-hyperlipidemia, anti-ulcer, anticancer, anti-inflammatory, antidiabetic, and antioxidant effects [9,11]. Studies on the effects of γ-oryzanol on the brain suggest its role in modulating the function of the hypothalamus and anterior pituitary gland [12–14]. Recently, γ-oryzanol has also been reported to preserve memory and cognitive functions in a rat model of sporadic Alzheimer’s disease [15]. However, the effect of γ-oryzanol on traumatic brain injury or SCI is unknown. To the best of our knowledge, this study is the first preliminary validation of the effects of γ-oryzanol in SCI. To this end, we intraperitoneally injected γ-oryzanol into mice with SCI and examined its effects using different methods, including behavioral tests.

## 2 Materials and methods

### 2.1 Animals

Female C57BL/6 mice (9 weeks old; average weight, 20 g) were purchased from the Comparative Medical Center of Yangzhou University (Yangzhou, China). Mice were housed under a 12 h light/dark cycle, 3–4 per cage, maintained at 20–22°C with 50% relative humidity, and provided water and food *ad libitum*.

### 2.2 Grouping and surgical procedures

Mice were randomly divided into three groups. Mice were anesthetized with 2% isoflurane in oxygen. The spinal cord was exposed with T10 as the center, and the left spinal cord was removed with the posterior midline of the spinal cord as the boundary, forming a 2 mm rectangular defect. A medical gelatin sponge was placed bilaterally in the paravertebral muscular space to absorb bleeding and prevent postoperative adhesion between the connective tissue and the dura mater. The muscles were repositioned and sutured, and the skin was stapled. The spinal cord of the sham group was exposed after left laminectomy without further injury.

### 2.3 Dose calculation

The dose for intraperitoneal injection of γ-oryzanol in mice was calculated as 100 mg/kg. This was based on the dose translation formula from humans to animals, which is based on the body surface area of the daily dosage of γ-oryzanol (MedChemExpress, China) of 500 mg/day in humans [16].

### 2.4 Postoperative treatment

To prevent postoperative infection, all mice with SCI were intramuscularly injected with ampicillin (150 mg/kg) once daily for 7 days, and the bladder was manually squeezed twice daily until the micturition reflex was restored. Mice in the sham group were only provided antibiotics. The injury group received saline injection intraperitoneally every 2 days, whereas the γ-oryzanol-treated group received 100 mg/kg γ-oryzanol intraperitoneally every 2 days.

### 2.5 Assessment of functional recovery of the spinal cord

Within 6 weeks of surgery, the recovery of right lower limb function was evaluated weekly using the Basso mouse scale (BMS) score and an oblique plate experiment to analyze the recovery process of motor function.

Gait analysis was performed at week 6 after SCI, the two hind plantar surfaces of each mouse were stained black using non-toxic ink, and the mice were allowed to crawl on a cut white paper track (5 × 15 cm^2^). The measures included stride length (distance between the centers of adjacent footprints on the same side) and sway distance (vertical distance between the center of the left and right hind limbs) and were statistically compared using the average of three steps for each condition.

### 2.6 Enzyme-linked immunosorbent assay (ELISA)

The levels of inflammatory factors in the peripheral blood of mice were assessed by ELISA 6 weeks after surgery. After fixation, the tails of the mice were immersed in hot water at 50°C for a few minutes to fill the tail blood vessels. The tail was dried, scissors were used to cut the tail tip to 1–2 mm, a test tube was used to connect the blood outflow, and the tail root to the tail tip was massaged. After blood collection, cotton ball pressure was applied to stop bleeding. In strict accordance with the manufacturer’s instructions, the levels of interleukin (IL)-6, tumor necrosis factor (TNF)-α, and IL-10 in the peripheral blood of mice were detected by ELISA using IL-6, TNF-α, and IL-10 ELISA test kits (BYabscience Co., LTD, China).

### 2.7 Damage area assessment

Magnetic resonance imaging (MRI) was performed on five animals in each group on day 42 after surgery. Mice were anesthetized with halothane (3–4% induction, 1.5–2% maintenance) in oxygen (0.4 L/min) and nitrogen (0.6 L/min). Anesthetized mice were placed on a fixation system in the prone position, and scans were performed using a small-animal MRI system (Bruker Biospec 11.7T animal MR scanner; Bruker AXS GmbH, Germany). The sequence protocol was performed using the following parameters: echo time, 23.000 ms; echo spacing, 7.667 ms; repetition time, 550.000 ms; repetitions, 1; average, 9; rare factor, 8; slice thickness, 1.000 mm; slice orientation, sagittal; read orientation, Ro-Cd.

Spinal cord hematoxylin and eosin (H&E) staining and Luxol fast blue (LFB) staining were performed 6 weeks after surgery. Mice were anesthetized with an intraperitoneal injection of 2% pentobarbital (50 mg/kg). T9–T11 spinal cord samples were rapidly dissected after cardiac perfusion with 4% paraformaldehyde and fixed with 4% (W/V) paraformaldehyde overnight. Tissues samples were dehydrated in a gradient of ethanol and embedded in paraffin. Paraffin-embedded tissues were cut into a thickness of 4 μm (Leica CM 1900, Germany) and subjected to H&E and LFB staining.

### 2.8 Immunofluorescence analysis

Tissues were processed as described in the aforementioned section, and 20 μm horizontal sections were cut on a cryostat. Spinal cord sections were washed thrice with 0.01 M phosphate-buffered saline (PBS) and incubated with primary antibodies overnight at 4°C. Subsequently, the spinal cord sections were washed thrice with PBS. The primary antibodies used in this study include mouse monoclonal anti-glial fibrillary acidic protein (GFAP) (1:200 dilution; Sigma-Aldrich, USA), to label the astrocytes; rabbit anti-mouse laminin antibody (1:200 dilution; Sigma-Aldrich); and rabbit anti-rat 5-hydroxytryptamine (5-HT) (1:200 dilution; Immunostar, USA), to label the motor neurons. The sections were then incubated with the corresponding secondary antibody for 1 h at room temperature protected from light. Finally, the sections were washed thrice with PBS and gently incubated with 4′,6-diamidino-2-phenylindole (DAPI) for 3 min protected from light. Excess DAPI was removed by washing with PBS, the slides were covered with glycerol and cover slips, and a field was randomly selected using a fluorescence microscope (BX-51; Olympus, Japan).

### 2.9 Statistical analysis

All experiments were repeated independently at least three times. SPSS software (version 22.0) (IBM, USA) was used for statistical analysis, and GraphPad Prism 8 software (GraphPad Software Inc., USA) was used to draw the graphs. Quantitative data were described as mean ± standard deviation (SD). Independent samples *t*-test was used for comparison between two groups, and one-way analysis of variance was used for comparison between multiple groups. Statistical significance was set at *P < 0.05, **P < 0.01, and ***P < 0.001.


**Ethical approval:** The research related to animals’ use has been complied with all the relevant national regulations and institutional policies for the care and use of animals. All the animal experiments were designed and approved by the Yangzhou University Laboratory Animal Ethics Committee (yzu-lcyxy-n060).

## 3 Results

### 3.1 Intraperitoneal γ-oryzanol injection restores motor function in SCI mice

**Figure 1:**
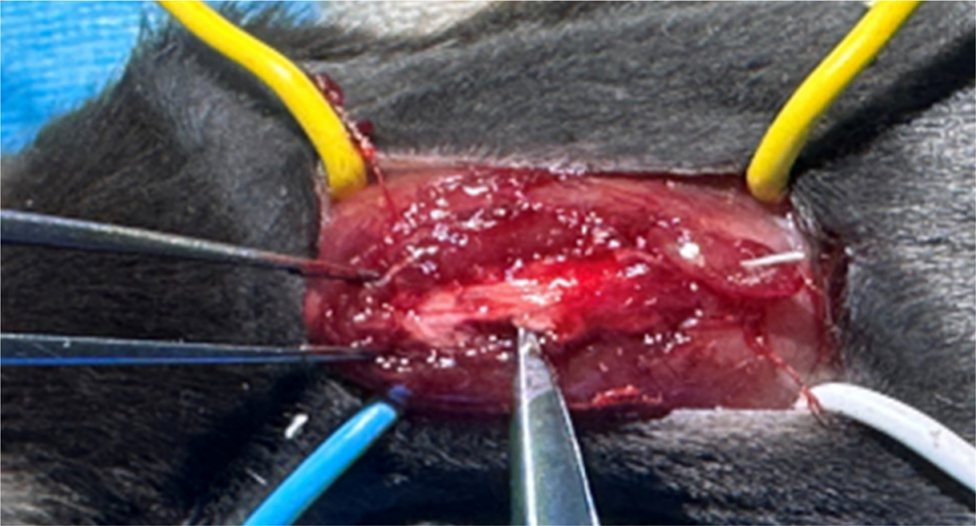
Protocol for SCI. The spinal cord of mice was exposed with T10 as the center and the left spinal cord was removed with the posterior midline of the spinal cord as the boundary, forming a 2 mm rectangular defect.

**Figure 2:**
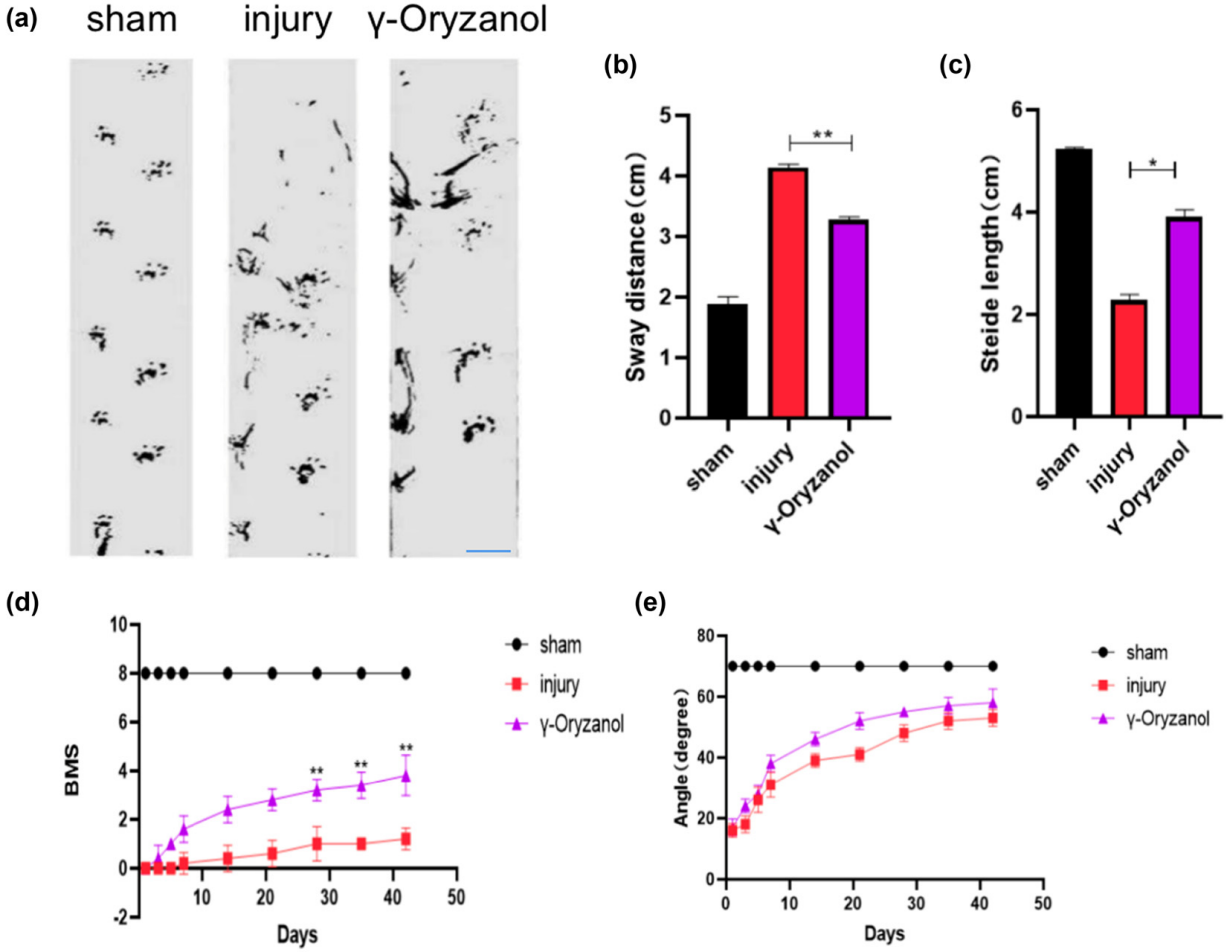
Intraperitoneal γ-oryzanol injection restored motor function in SCI mice: (a) Representative footprint patterns of the three groups 6 weeks after SCI. Scale bar = 2.5 cm; (b) and (c) semiquantitative analysis of stride length (b) and sway distance (c) in the gait analysis; (d) BMS score of mice 6 weeks after SCI; and (e) slant plate experiment score of mice 6 weeks after SCI. Data are presented as mean ± SD (*n* = 5). **P < 0.01 and *P < 0.05.

SCI was modeled in mice as described previously (Figure 1). The restoration of motor function is the most important measure of SCI repair. Six weeks after surgery, mice in the sham group showed a strong and stable normal gait during gait analysis, whereas mice in the injury group showed severe toe dragging, increased swing distance (4.14 ± 0.04), and reduced stride length (2.28 ± 0.09). On the other hand, the stride, swing distance (3.28 ± 0.04), and stride length (3.90 ± 0.12) of mice in the γ-oryzanol-treated group were close to those of the sham group (Figure 2a–c). Additionally, motor behavior was assessed using the BMS score, and the results showed that both the SCI and γ-oryzanol-treated groups showed significant left hindlimb paralysis from days 1 to 7 after injury. However, from week 4 to week 6 post-trauma, the BMS scores improved considerably in the γ-oryzanol-treated group compared to that in the injury group (Figure 2d). In the oblique plate experiment, although the γ-oryzanol-treated group showed a higher response than the injury group, the difference was not statistically significant (Figure 2e).

### 3.2 Intraperitoneal γ-oryzanol injection reduces the damage area in SCI mice

**Figure 3:**
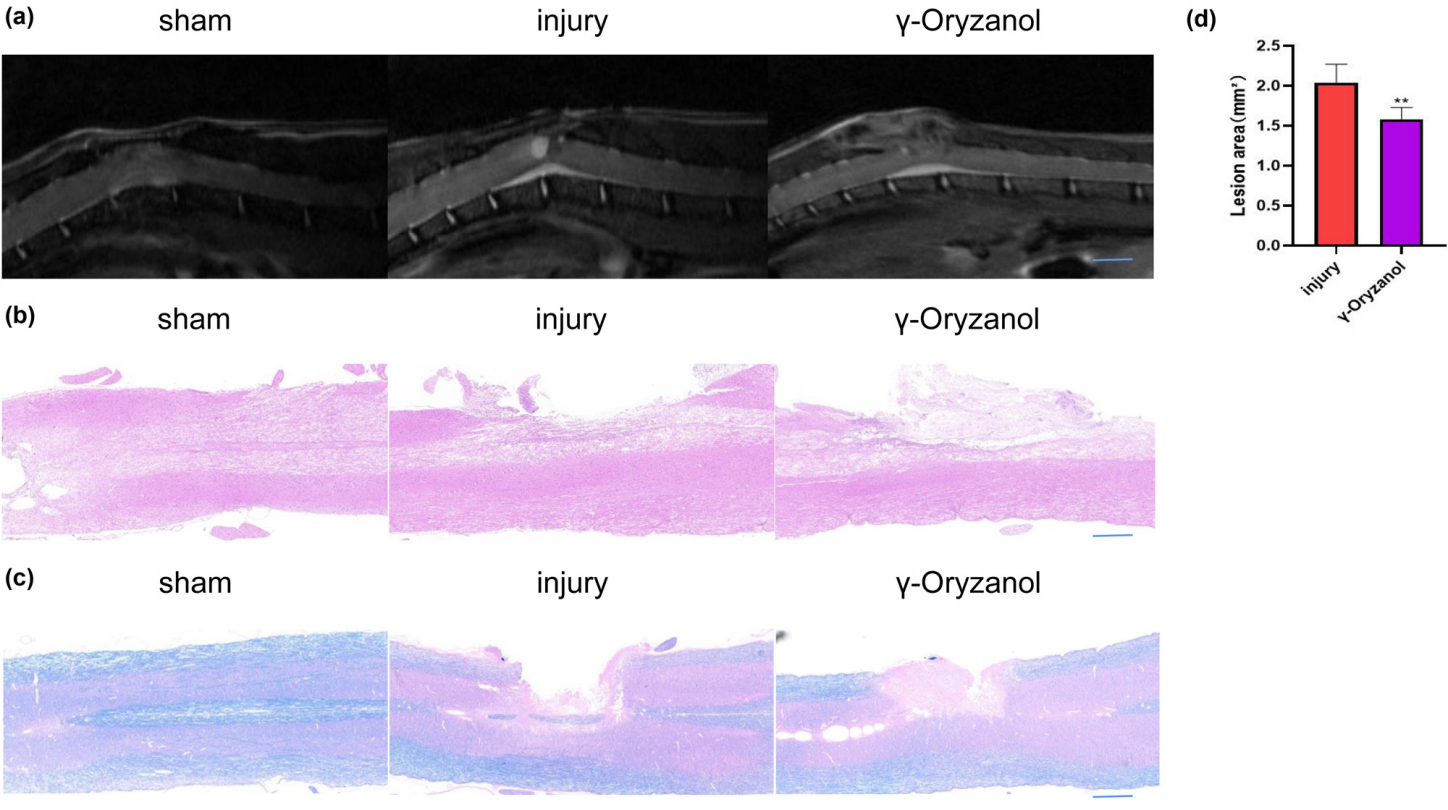
Intraperitoneal γ-oryzanol injection reduced the damage area in SCI mice: (a) Representative sagittal and coronal MRI images. Scale bar = 1 mm; (b) and (c) spinal cord tissue with H&E (b) and LFB (c) staining; and (d) quantitative analysis of the lesion area. Data are presented as mean ± SD (*n* = 5). **P < 0.01.

To verify the *in vivo* neuroprotective effects of intraperitoneal γ-oryzanol injection, we performed MRI of the thoracic spinal cord (T10) in mice. MRI showed that intraperitoneal γ-oryzanol injection (1.58 ± 0.13) reduced the lesion area compared to that in the injury group (2.04 ± 0.20) (Figure 3a and d). H&E staining also showed similar results; considerable spinal cord tissue loss was observed in the injury group 6 weeks after injury. However, the γ-oryzanol-treated group exhibited a considerable reduction in the area of tissue loss 6 weeks after injury (Figure 3b). Similar results were observed with LFB staining with respect to the protection of the myelin sheath (Figure 3c).

### 3.3 γ-Oryzanol treatment suppresses neural scar formation and 5-HT neuron loss in SCI mice

**Figure 4:**
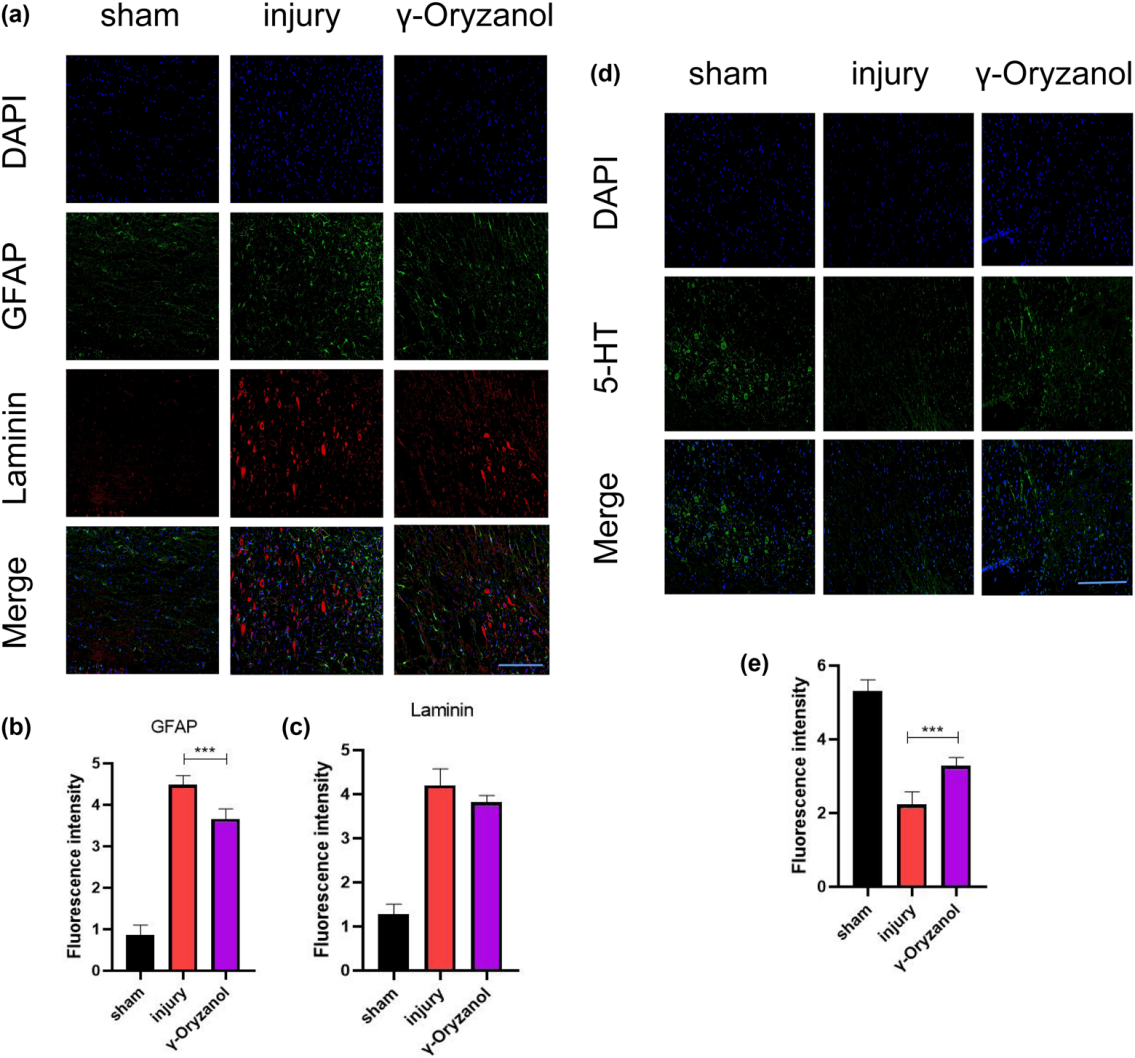
γ-Oryzanol treatment suppressed neural scar formation and 5-HT neuron loss in SCI mice: (a) Analysis of the inhibition of glial scar formation by double immunofluorescence labeling of GFAP (green) and laminin (red) after 6 weeks of treatment. Nuclei were stained with DAPI (blue). Scale bar = 50 μm; (b) and (c) quantitative analysis of fluorescence intensity of GFAP- (b) and laminin- (c) positive cells (c); (d) analysis of axon growth by immunofluorescence labeling of 5-HT (green) after 6 weeks of treatment. Nuclei were stained with DAPI (blue). Scale bar = 50 μm; and (e) quantitative analysis of the fluorescence intensity of 5-HT-positive cells. Data are presented as mean ± SD (*n* = 5). ***P < 0.001.

The formation of glial scar and fiber scar after SCI is not conducive to the regeneration and repair of the spinal cord. To understand the effect of intraperitoneal γ-oryzanol injection on the growth of nerve scars, spinal cord tissue samples were double-stained for GFAP and laminin. As shown in Figure 4, GFAP expression was lower in the γ-oryzanol-treated group (3.66 ± 0.21) than that in the injury group (4.48 ± 0.20) (Figure 4a and b). It is noteworthy that although laminin expression in the γ-oryzanol-treated group (3.82 ± 0.13) was lower than that in the injury group (4.60 ± 0.33), the difference was not significant (Figure 4a and c). Additionally, the intensity of 5-HT axons is an important indicator of the degree of retention of response motor function. The intensity of 5-HT axons (3.28 ± 0.20) in the γ-oryzanol-treated group was closer to that in the sham group (5.30 ± 0.28) (Figure 4d and e), indicating the retention of motor function.

### 3.4 Intraperitoneal γ-oryzanol injection reduces the inflammatory response in SCI mice

**Figure 5:**
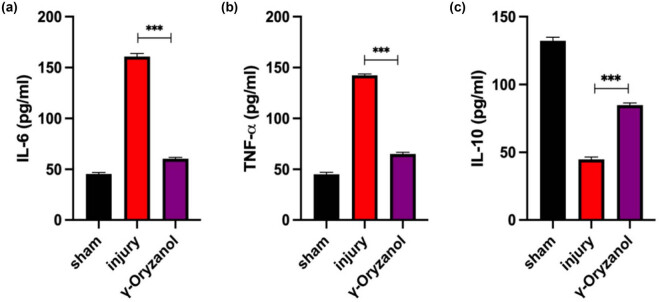
Intraperitoneal γ-oryzanol injection reduced the inflammatory response in SCI mice: (a)–(c) ELISA analysis of IL-6 (a), TNF-α (b), and IL-10 (c) levels in the peripheral blood serum of SCI mice. Data are presented as mean ± SD (*n* = 5). ***P < 0.001.

Furthermore, we investigated the effect of γ-oryzanol on inflammation in SCI mice. To this end, the levels of IL-6, TNF-α, and IL-10 in the peripheral blood serum of SCI mice were measured by ELISA. As shown in Figure 5, compared with the sham group, the levels of IL-6 (160.82 ± 5.82) and TNF-α (142.35 ± 5.77) were considerably increased in the injury group, while the levels of IL-10 (44.77 ± 3.71) were considerably decreased. However, γ-oryzanol treatment decreased the levels of these pro-inflammatory factors (IL-6 and TNF-α), but increased the level of IL-10 (84.79 ± 3.41). These data suggest that γ-oryzanol mitigates the systemic inflammatory response in mice after SCI.

## 4 Discussion

Post-traumatic SCI recovery is challenging due to poor neurological recovery and limited clinical response as the current clinical therapies have yet to achieve satisfactory results [17–19]. γ-Oryzanol can reportedly cross the blood–brain barrier in an intact form and is highly distributed in the brain. γ-Oryzanol exerts various biological effects including antioxidant, anti-tumor, anti-diabetic, anti-ulcer, neuroprotective, and immunomodulatory effects [20,21]. Furthermore, γ-oryzanol is a popular energy adjuvant that has been shown to be safe with no major side effects in animals and humans and is hence registered in the US Food and Drug Administration System (UNII SST9XCL51M) (http://fdasis.nlm.nih.gov/srs/). γ-Oryzanol emulsion can be purchased as a dietary supplement for humans and animals, such as dogs and horses [22]. In the present study, we are the first to report that γ-oryzanol may effect motor function recovery after SCI in mice.

Abnormal gait can be attributed to reduced muscle tone, cerebellar movement, impaired peripheral nerve function, or underlying musculoskeletal abnormalities [22]. In the present study, the sham group showed a stable and strong normal gait, whereas decreased stride size and increased steps were observed in the injury group 6 weeks after injury, which suggests severe movement impairment. The γ-oryzanol group exhibited some degree of gait recovery; however, the degree of gait recovery varied, which may be due to the different degrees of regeneration of conduction tracts and nerve cells in white and gray matter. Inconsistent muscle tension caused by the atrophic lower limb muscles was regularly monitored to assess the recovery of motor function in SCI mice. To this end, we assessed BMS scores at scheduled time points. Except for the sham group, left hind limb paralysis was noted immediately after surgery, with a BMS score close to 0. In the injury group, the BMS score did not exceed four points after 6 weeks of injury, indicating that no compensatory recovery occurred after SCI. In the γ-oryzanol-treated group, the BMS score gradually increased over time, and from week 3 after surgery, mice in the γ-oryzanol-treated group had significantly higher BMS scores than in the injury group.

To obtain a comprehensive view of the repair of spinal cord tissue, spinal MRI and H&E staining and LFB staining of spinal cord tissue samples were performed. MRI showed that γ-oryzanol injection reduced the lesion area compared with that in the injury group. Mice were sacrificed 6 weeks after surgery, and the spinal cord was completely removed. Importantly, we observed new tissue growth at the site of injury in all groups. However, because the damage area is only about 2 mm, it is difficult to distinguish the area to be observed. Therefore, we further performed H&E staining. The injury group did not exhibit tissue growth, contrary to what was observed grossly. This could be attributed to the obstruction of other surrounding tissues during gross observation. The γ-oryzanol-treated group had reduced cavity area and new tissue compared to the injury group. Meanwhile, compared with the sham group, the SCI and γ-oryzanol-treated groups exhibited a certain degree of demyelination after injury. While the injury group exhibited no obvious remyelination, in the γ-oryzanol-treated group, remyelination was observed. Collectively, these results suggest that γ-oryzanol may promote the growth and infiltration of nerve cells and tissue recovery.

Astrocytes are the main type of glial cells in the central nervous system, and their sustained functional activity after SCI can lead to excessive astrocyte proliferation and glial scar formation, which is unfavorable for spinal cord regeneration. Laminin-rich fibrous scar tissue formation at lesion sites after SCI is a key barrier to axon regeneration as the glial scar is rich in GFAP [8]. To assess nerve scar formation, double staining for GFAP and laminin was performed. GFAP expression in the γ-oryzanol-treated group was significantly lower than in the injury group. Interestingly, the expression of laminin was not significantly different between the two groups, which we would like to explore in the future. 5-HT is a marker of axons of motor neurons. 5-HT expression was significantly higher in the γ-oryzanol-treated group than that in the injury group. These results suggest that γ-oryzanol is a drug with potential neurotrophic activity and may facilitate the retention of motor function to some extent.

γ-Oryzanol reportedly exerts immunomodulatory effects [20,21]. Hence, we evaluated the levels of pro-inflammatory factors in the peripheral blood serum of mice with SCI by ELISA. γ-Oryzanol treatment decreased the expression of the pro-inflammatory factors IL-6 and TNF-α but increased IL-10 levels. This suggests that γ-oryzanol may play a role in regulating systemic inflammatory responses in SCI. In the future, we aim to investigate the mechanism underlying the immunomodulatory effects of γ-oryzanol and its association with various inflammatory cells and factors.

The present study has some limitations. γ-Oryzanol has been widely used in humans, and although many animal experiments refer to the human dosage, the optimal dosage for rodents has not been determined. Additionally, intraperitoneal injection, although simple to administer, may not be the most effective method. We aim to explore more suitable drug delivery methods for the treatment of SCI. Additionally, γ-oryzanol may preserve axons through its immunomodulatory effects. However, the effects of γ-oryzanol on various inflammatory cells involved in the systemic immune response in SCI and its effects on axonal regeneration remain unknown.

In the future, we aim to investigate the mechanism underlying the immunomodulatory effects and axon generation capacity of γ-oryzanol after SCI. Additionally, we aim to elucidate the effect of γ-oryzanol on various inflammatory cells and inflammatory factors and whether it regulates axon generation through the modulation of inflammatory cells as well as the underlying mechanism. If our hypothesis holds, we aim to conduct a clinical trial to assess the best method of administration of γ-oryzanol and to determine the optimal drug concentration corresponding to the different methods. Because of its high safety and low price, γ-oryzanol is a promising candidate for the clinical treatment of SCI.

## 5 Conclusion

γ-Oryzanol is a natural compound that is easy to extract and is relatively inexpensive. Our results suggest that intraperitoneal injection of γ-oryzanol can improve motor function and reduce the area of SCI in mice. Additionally, γ-oryzanol treatment facilitated the retention of 5-HT neurons and decreased glial scarring. γ-Oryzanol treatment also decreased the serum levels of IL-6 and TNF-α and increased the serum levels of IL-10. Our findings suggest that downregulation of the systemic inflammatory response may facilitate the recovery of motor function following SCI and that γ-oryzanol is a promising treatment approach for SCI.
